# Demonstration of ion channel synthesis by isolated squid giant axon provides functional evidence for localized axonal membrane protein translation

**DOI:** 10.1038/s41598-018-20684-8

**Published:** 2018-02-02

**Authors:** Chhavi Mathur, Kory R. Johnson, Brian A. Tong, Pablo Miranda, Deepa Srikumar, Daniel Basilio, Ramon Latorre, Francisco Bezanilla, Miguel Holmgren

**Affiliations:** 1 National Institute of Neurological Disorders and Stroke, National Institutes of Health, Bethesda, Maryland 20892 USA; 2 Bioinformatics Section, National Institute of Neurological Disorders and Stroke, National Institutes of Health, Bethesda, Maryland 20892 USA; 3 Facultad de Ciencias, Universidad de Chile, Santiago, 7750000 Chile; 4 Centro Interdisciplinario de Neurociencias de Valparaíso, Universidad de Valparaíso, Valparaíso, 2366103 Chile; 5 Department of Biochemistry and Molecular Biology, University of Chicago, Gordon Center for Integrative Sciences, Chicago, Illinois 60637 USA

## Abstract

Local translation of membrane proteins in neuronal subcellular domains like soma, dendrites and axon termini is well-documented. In this study, we isolated the electrical signaling unit of an axon by dissecting giant axons from mature squids (*Dosidicus gigas*). Axoplasm extracted from these axons was found to contain ribosomal RNAs, ~8000 messenger RNA species, many encoding the translation machinery, membrane proteins, translocon and signal recognition particle (SRP) subunits, endomembrane-associated proteins, and unprecedented proportions of SRP RNA (~68% identical to human homolog). While these components support endoplasmic reticulum-dependent protein synthesis, functional assessment of a newly synthesized membrane protein in axolemma of an isolated axon is technically challenging. Ion channels are ideal proteins for this purpose because their functional dynamics can be directly evaluated by applying voltage clamp across the axon membrane. We delivered *in vitro* transcribed RNA encoding native or *Drosophila* voltage-activated Shaker K_V_ channel into excised squid giant axons. We found that total K^+^ currents increased in both cases; with added inactivation kinetics on those axons injected with RNA encoding the Shaker channel. These results provide unambiguous evidence that isolated axons can exhibit *de novo* synthesis, assembly and membrane incorporation of fully functional oligomeric membrane proteins.

## Introduction

Neurons are morphologically compartmentalized cells. Their cell bodies possess the nucleus and organelles to synthesize proteins, many of which are transported to axons and dendrites. Nonetheless, cumulative evidence has suggested that local protein translation is essential^1–3^ for dendritic function^4–13^, axonal maintenance^14,15^, as well as for external stimuli-induced responses in specialized axonal structures, such as in growth cones during axonal development^16–20^ and navigation^15,21–24^, and in axon termini during axonal regeneration^25,26^ and synaptic plasticity^27^. Consistently, large number of mRNA transcripts have been reported in these compartments^28–36^.

**Figure 1:**
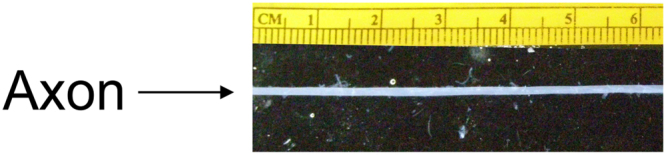
Actual size photograph of an axon’s segment of the squid *Dosidicus gigas*. Size estimation using a metric scale (top).

Using genomewide microarrays or next-generation sequencing with pure axonal preparations^30,33,35^, the number of unique transcripts in axons are now believed to be in the thousands; and likely many are actively translated locally^37^. In this study, we aimed to obtain a transcriptome using axoplasm, exclusively derived from the giant axon of the Humboldt squid (*Dosidicus gigas*). These axons are known to be formed by fusion of axons originating from cell bodies residing in the stellate ganglion^38^ that gives rise to a large electrical signal transmission unit, normally larger than 1.0 mm in diameter (Fig. 1). Axoplasm was separated from axolemma and surrounding Schwann cells to assess the population of RNA transcripts present in one single axon. Total RNA from axoplasm of squid giant axons was found to contain ribosomal RNAs (rRNAs) and messenger RNAs (mRNAs), which support axonal protein translation. These axons also showed an enriched population of signal recognition particle (SRP) RNA and mRNAs encoding protein subunits that are known to bind this RNA molecule to form SRP complex, required for co-translational transport to ER of nascent polypeptide chains encoding membrane and secretory proteins^39,40^. Previously reported transcriptomes^30,33^, as well as the translatome obtained from axons^37^, have shown that they contain mRNA encoding membrane and secreted proteins, as well as proteins involved in their biosynthesis. In agreement, imaging studies have indicated plausible presence of endomembrane components and locally synthesized membrane proteins in cultured mammalian axons^41–43^.

Synthesis of membrane proteins such as ALCAM (activated leukocyte cell adhesion molecule)^20^, receptor EphA2 (ephrin receptor A2)^21^, NFPC (NF-protocadherin)^24^ in axon growth cones has been found to influence axon navigation and growth in cell culture, suggesting that these locally synthesized proteins are functional. Similarly, Spencer *et al*.^44^ have shown that local translation of conopressin receptor in *Aplysia* axons could alter the membrane excitability properties in response to externally added conopressin. In the present study, we examined whether membrane proteins, newly synthesized by isolated squid giant axons, are functional. Because *Dosidicus*’ axons have a large diameter, we can utilize them for direct RNA delivery inside the axoplasm. Thus, we injected squid giant axons with *in vitro* synthesized RNA encoding heterologous *Drosophila* Shaker K_V_ channel or native *D. gigas* K_V_1.1. We then monitored the incorporation of newly synthesized ion channels into axon membrane by voltage clamp, which allowed us to study directly, the function of these membrane proteins. In both cases we found an increase in potassium currents across the axolemma. Furthermore, we observed that local synthesis of functional heterologous Shaker K_V_ channel, which inactivates with fast kinetics, carried new discernable functional properties to the squid axon system. Since K_V_ channels are known to undergo post-translational processing and oligomerization in endomembranous compartments prior to membrane-targeting^45,46^, our study unambiguously demonstrated that new K_V_ channel subunits can be *de novo* translated, folded and hetero-tetramerized into functional ion channels that were targeted to axolemma likely using the axoplasm’s resident endomembranous machinery.

## Results

### Transcriptome analysis of extruded axoplasm from squid giant axons

**Figure 2:**
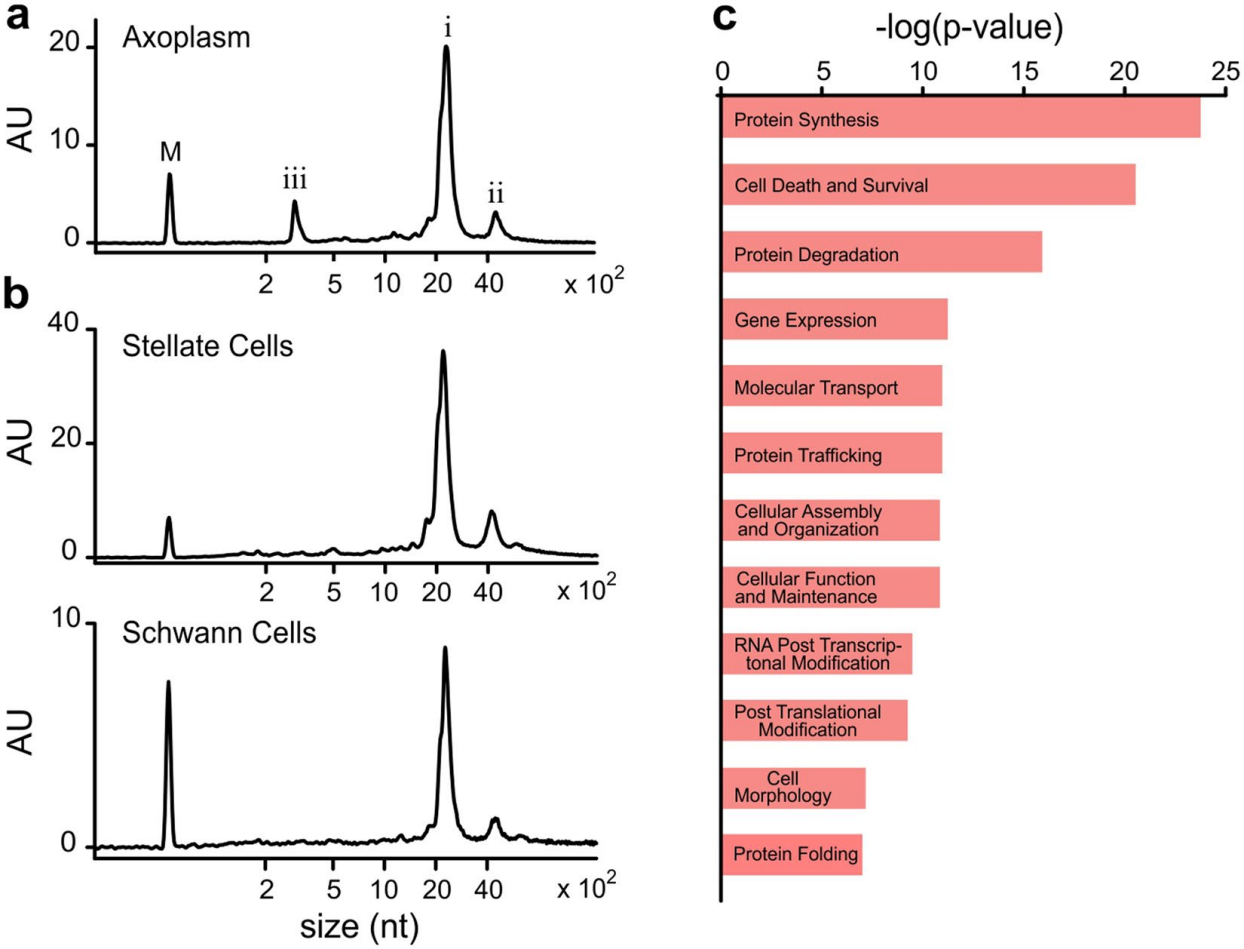
RNA composition of *Dosidicus gigas* giant axon system. (**a**) Representative bioanalyzer (Agilent 2000) profile of total RNA from squid giant axoplasm. Peak (i; ~2,300 nt) corresponds to 18 S rRNA and the 28 S rRNA fragments^70^. Peak (ii) corresponds to ~4400 nt unfragmented 28 S rRNA. Peak (iii) corresponds to an axoplasm’s enriched RNA species of ~300 nt. M: RNA marker (25 nt). (**b**) Profile of total RNA from cell bodies within the squid giant axon system. (**c**) Functional annotation of most abundant axonal ORFs (top 25^th^ percentile RPKM) presented in decreasing order of significance. Here are shown only the twelve-most highly significant categories.

Deep sequencing studies of RNA species resident in mammalian axons have suggested that the local protein synthesis occurs extensively in axons, and have thus opened new avenues to study their functional regulation. Squid giant axons, due to their size, ease of manipulation and similarity in function with mammalian neurons, offered us a system for analyzing axonal protein translation at the resolution of a single axon. So far, a full-scale unbiased assessment of the RNA composition of squid giant axons has remained unknown. In our experiments, good quality RNA was obtained in microgram quantities from the axoplasm extruded from a single axon. Figure 2a shows the profile of total RNA isolated from the axoplasm of *D. gigas*. For comparison, bioanalyzer profiles of total RNA from Stellate ganglion which contains the cell bodies of the giant axon, and Schwann cells which surround the giant axon are shown in Fig. 2b. Peaks (i) and (ii) in all bioanalyzer profiles correspond to rRNA which was found to be the most abundant RNA species in both axons and cell bodies. The mRNA content of the axons was specifically analyzed by constructing poly(A)-enriched cDNA libraries (Illumina Truseq) from *D. gigas* axoplasm, and subjecting them to deep sequencing (RNA-Seq) using Hi-Seq. 2000 RNA-seq platform (Illumina). Upon performing quality control, ~95% reads were found to have a PHred score > 30. Since the reference genome sequence for *D. gigas* is not available, sequence reads from the RNA-seq were used to perform a *de novo* assembly to generate sequence contigs, referred to as components, which would represent the “squid axonal transcriptome”. This assembly generated 86,518 sequence components with N50 length of 756 nts and ~40% GC content. For each component generated, ORFs were predicted and blastx characterized against Swiss-prot database allowing the identification of open reading frames (ORFs) corresponding to 7820 unique genes (Supplementary Table S1). Axoplasm’s RNA-Seq reads were mapped back to the components obtained from the axonal *de novo* assembly. Numbers of mapped reads per kilobase per million reads (RPKM) were used to estimate the relative abundance of individual axoplasmic ORFs (Supplementary Table S1).

A large proportion of the mRNA present in the axons was found to encode for ribosomal subunits, elongation factors, cytoskeletal, metabolic and heat shock proteins (Supplementary Table S1). The ORFs with RPKM within the top 25^th^ percentile were mapped to the functional analysis categories in Ingenuity Pathway Analysis. Approximately, 73% ORFs were mapped to 28 distinct molecular pathways, of which the top twelve are shown in Fig. 2c. Amongst these categories, protein synthesis was found to be the most highly represented cellular process (Fig. 2c). The squid axonal transcriptome was also found to contain 483 mRNAs encoding for proteins that are known to localize to the plasma membrane and 41 to the extracellular matrix (Supplementary Table S1; yellow and orange, respectively). This list includes transporters, ion channels, adhesion proteins, membrane proteases, basement membrane proteins and collagen. The capability to synthesize membrane and secretory proteins suggested the presence of rough ER and the Golgi apparatus in axons, although the presence of both these organelles in mammalian axons is currently controversial^41,42^. In squid axons, 134 mRNAs were found which encode for proteins involved in ER and Golgi function and structure, such as translocon, protein disulphide isomerase, O-linked N-acetylglucosamine hydrolase, receptor expression-enhancing protein 5, Golgin family of proteins (Supplementary Table S1; green). These results ascertained that many transcripts that encode for members of the molecular machinery needed for membrane protein synthesis are present in squid giant axons.

### Signal recognition particle components in axoplasm

**Figure 3:**
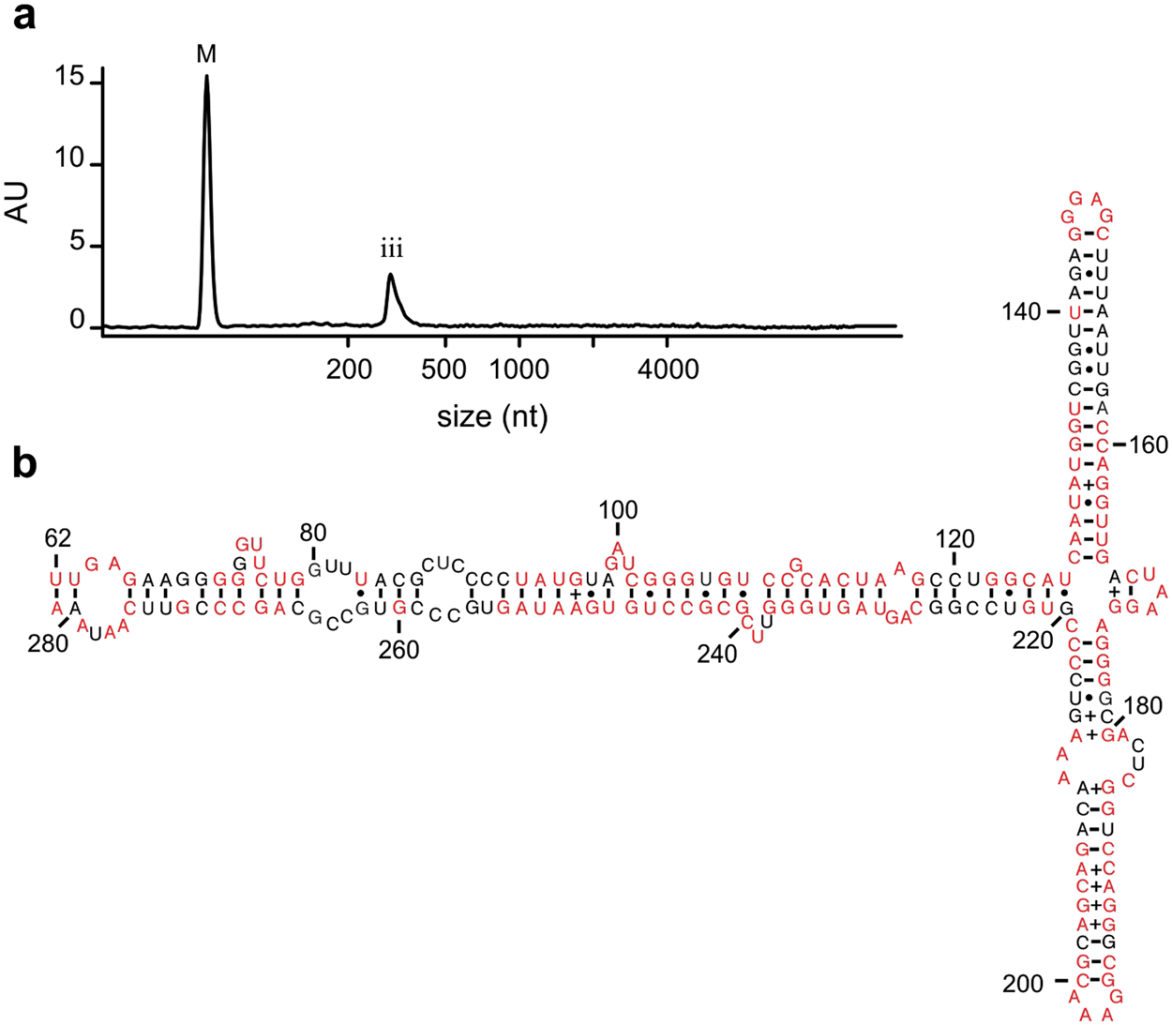
Characterization of the ~300 nt long RNA species in *Dosidicus gigas* giant axoplasm. (**a**) Enrichment of the ~300 nt RNA species. Bioanalyzer profile of axonal RNA after rRNA depletion from giant axon total RNA shows a substantial enrichment of peak (iii). M: RNA marker (25 nt). (**b**) Predicted secondary structure of the large S domain (62–281 nts) *for Dosidicus gigas* signal recognition particle (SRP) RNA. Conserved and deviant nucleotides are represented in red and black, respectively.

**Figure 4:**
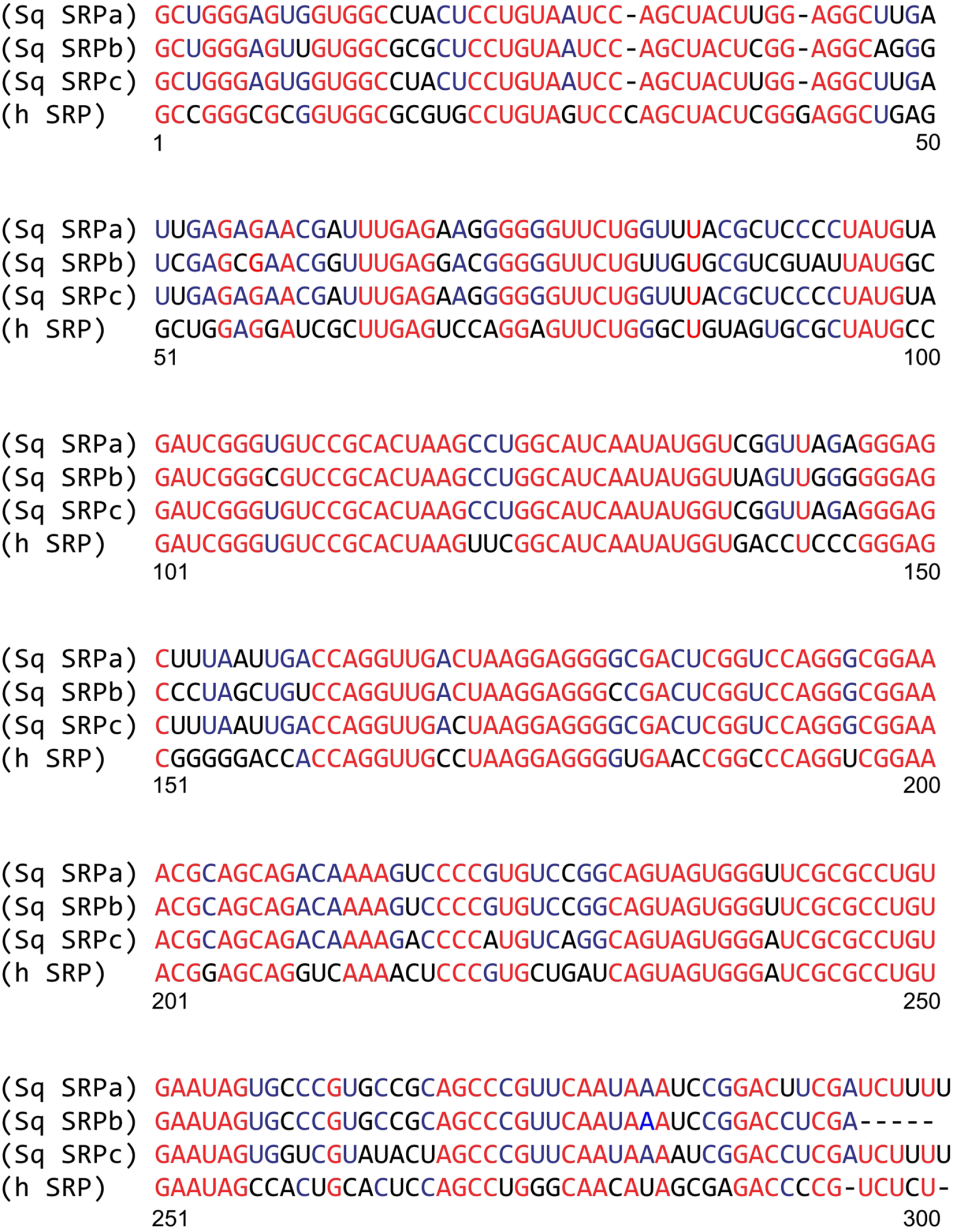
Squid SRP RNA alignment. De novo assembly of rRNA-depleted axon sample generated sequence components encoding Sq SRP RNA a, b and c which represented 38, 23 and 4 percent of all mapped back reads. The independent identities with human SRP RNA are between 67.4 and 68.6%. Nucleotides conserved in all sequences are represented in red, nucleotides conserved in all squid sequences in blue, and non-conserved nucleotides in black.

Notably, the axon’s RNA composition presented a unique peak (iii) at ~300 nt in the bioanalyzer profile (Fig. 2a); unlike the total RNA profiles from the cell bodies of the giant axon and the Schwann cell layer that surrounds the axolemma (Fig. 2b). However, in the poly(A)-based transcriptome assembly, a ~300 nt long component with a significantly higher RPKM value that might be representative of the RNA species under peak (iii) was not observed. This result suggested that the ~300 nt long RNA species is likely a non-coding RNA. To enrich the ~300 nt RNA species, we depleted rRNA from the *D. gigas* axon sample (Fig. 3a). This sample was used to prepare random primed cDNA library (Truseq Stranded Total RNA with Ribo-Zero Globin kit (Illumina)) as described in the Methods section. RNA-Seq data was collected and *de novo* assembled. Reads were then mapped back to the components generated and RPKMs were calculated. The first three components with the highest RPKM accounted for 38%, 23% and 4% of all the reads mapped back. These components were blastn characterized and were found to be similar in sequence to the ~300 bp long Signal Recognition Particle (SRP) RNA. These sequences (SRPa, SRPb and SRPc, respectively) upon alignment to human SRP (hSRP) RNA (Fig. 4) showed 67.4–68.6% identity to hSRP RNA. The sequence for SRPa was mapped back to the secondary structure of hSRP^47^. The large S domain (62–281 nts; Fig. 3b) was found to be highly homologous. This domain has been reported to recruit SRP68, SRP72, SRP54 and SRP19 proteins to form a nucleoprotein complex called SRP. SRP has been shown to recognize a nascent polypeptide chain, bound to ribosome, of a protein that would be targeted to the ER^39^. SRP has been demonstrated to then dock onto the SRP receptor (SR) present on the ER membrane which would interact with the Sec61 complex (translocon) to translocate the protein into the ER co-translationally, in a GTP-dependent manner. The squid axonal transcriptome generated in this study was found to contain mRNAs encoding SRP54, SRP68 and SRP72 which bind to SRP RNA, as well as the ER resident membrane proteins SRα, SRβ and Sec61α (Supplementary Table S1). While cell bodies would also contain SRP components, the enrichment of SRP RNA in axons was further indicative of plausible extensive membrane protein synthesis which would be critical for axonal function.

### Membrane expression of potassium channels in squid giant axons

**Figure 5:**
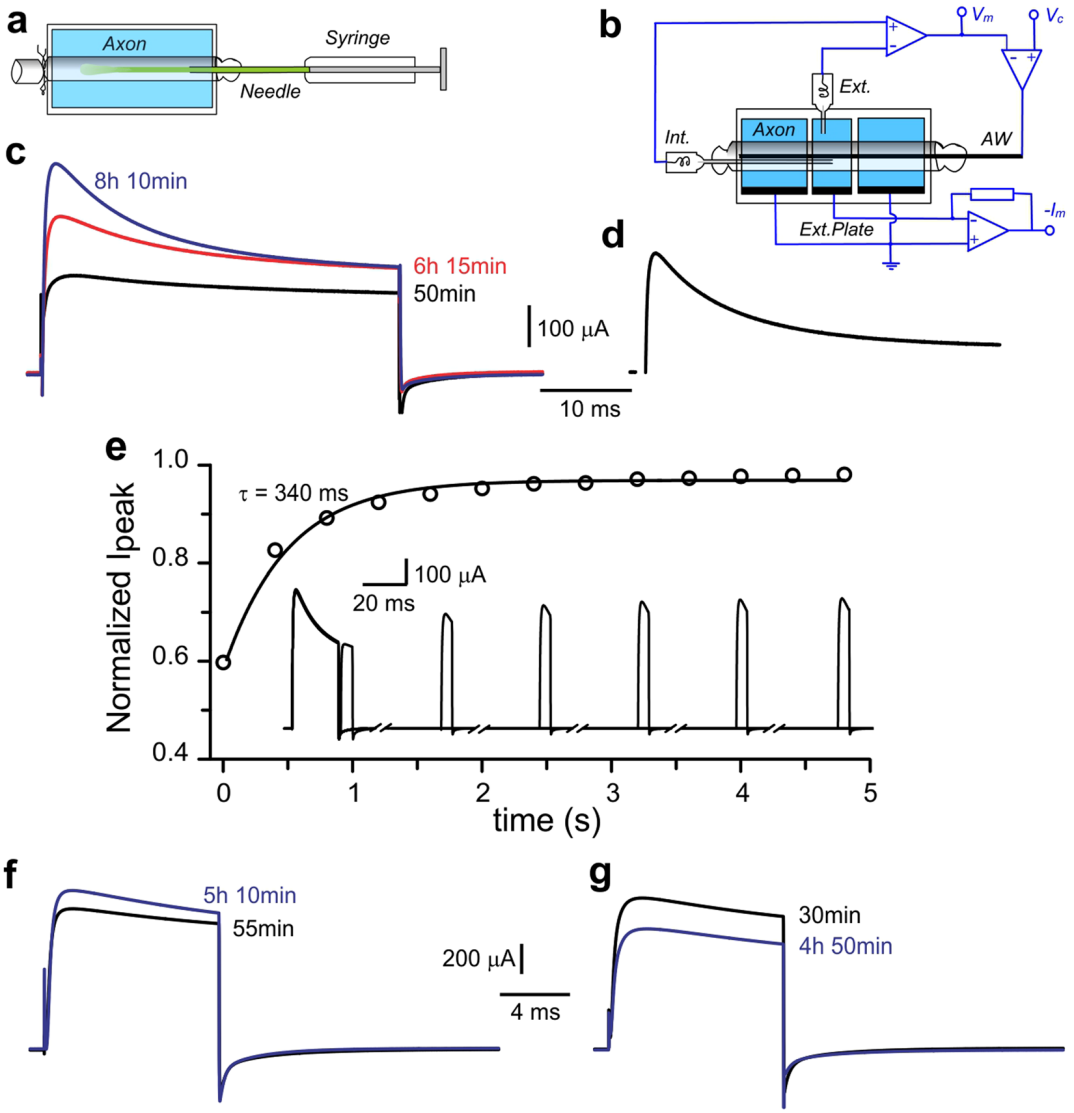
Membrane protein synthesis in isolated axons. (**a)** Axons were injected with a 230 μm diameter needle, as shown. (**b**) Electrophysiological set-up. Voltage clamp of the axolemma is achieved by passing current between the axial wire (AW) and the external plates (Ext. Plate) along the middle compartment of the chamber. Negative feedback clamps the membrane voltage V_m_ between an internal electrode (Int.) in the middle compartment and an external electrode (Ext.) outside of the axon to the command voltage V_c_. (**c)** Shaker K_V_ channel incorporation into axonal membrane. Potassium currents in response to a voltage step from −70 mV to + 80 mV recorded 50 minutes (black), 6 hours 15 minutes (red) and 8 hours 10 minutes (blue) after the injection of cRNA encoding for Shaker K_V_ channels. (n = 7). (**d**) Newly synthesized axonal potassium channels. Potassium current increment observed by subtracting the first recording from the last measurement acquired. (**e**) Recovery from inactivation of newly synthesized K_V_ channels. Inset shows six paired-pulse current recordings. Activating pulses were to + 70 mV, 20 ms and test pulses were 5 ms long. Recovery was assessed at −70 mV. Broken time periods: 360 ms. Single-exponential fit τ = 340 ms (solid line). (**f**) Potassium currents in response to a voltage step from −70 mV to + 80 mV recorded from an axon injected with cRNA encoding for Sq.K_V_1.1 channels. Black trace, 55 min. after cRNA injection. Newly synthesized *D. gigas* K_V_1.1 channels incorporated into the axolemma, increased the current (blue trace). (n = 2). (**g**) Axon’s endogenous potassium currents. Endogenous potassium currents in axons buffer-injected without cRNA (black trace) decay over time (blue trace). (n = 2).

To validate that the molecular machinery for membrane protein translation within squid giant axons is functional, we examined whether it would be feasible to detect incorporation of a newly synthesized membrane protein in the axon membrane by monitoring for its characteristic functional properties. As a proof of principle for the squid giant axon system, we chose to inject axons with *in vitro*-transcribed cRNA encoding for Shaker K_V_ channel. In addition to being an exogenous membrane protein for squid, these channels are known to display a distinctive and unique fast inactivation which is absent in squid K_V_ channels expressed natively. cRNA was injected along the axon’s length with a Hamilton syringe (0.5 μl) supplied with a needle gauge 32 (Fig. 5a). After injection, axons were mounted in an electrophysiological chamber (Fig. 5b). Since the electrical currents were measured between an electrode inside the axon (*AW*) and one outside (*Ext. Plate*) they would represent the ionic current that originates exclusively at the axolemma under voltage controlled between the inside (*Int*.) and outside (*Ext*.) of the axon. Figure 5c shows in black a K^+^ current recording obtained about 50 min. after cRNA injection, which was the approximate time required to mount an axon in the voltage clamp chamber. The ionic current observed was in response to a voltage step to + 80 mV from a holding potential of −70 mV. The characteristics of this initial K^+^ current were indistinguishable from those carried by native squid K_V_ channels (Fig. 5g). Because −70 mV is the approximate reversal potential for K^+^, there should have been no tail currents at the OFF step. However, this was not the case due to the accumulation of permeating K^+^ in the Frankenhaeuser-Hodgkin space between the axolemma and the Schwann cells. As a result, the reversal potential for K^+^ was found to be shifted more positively and consequently tail currents develop at −70 mV^48^. Remarkably, in a few hours after cRNA injection K^+^ currents were found to increase substantially and began showing signs of inactivation (Fig. 5c; red and blue traces). As time progressed, the relaxation decay of the inactivation process was seen to become faster. This observation can be most parsimoniously attributed to incorporation of new axonally translated K^+^ channel subunits containing inactivation particles into the axolemma (Fig. 5c). An estimate of the K^+^ currents from newly synthesized channels can be obtained by subtracting the initial K^+^ currents from the current recorded after ~7 hours (Fig. 5d**)**. In contrast to the initial current, these new channels were found to exhibit a profound fast inactivation process. As expected, those channels that inactivated, were seen to recover from it with a relatively slow time constant **(**Fig. 5e). All these observations can only be explained by newly synthesized Shaker K_V_-like channels being added to the axon membrane. Additional support for the hypothesis that membrane proteins can be synthesized *de novo* in the axon was obtained by injecting into axons cRNA encoding for *D. gigas* K_V_1.1 channels. In this case, K^+^ current corresponding to the overexpressed Squid K_V_1.1 channels were found to increase by ~15% within 5 hours (Fig. 5f), while endogenous K^+^ currents are known to normally rundown in that period of time, as also seen upon mock-injection of axons with the buffer alone (Fig. 5g).

### Hetero-oligomerization of potassium channels

**Figure 6:**
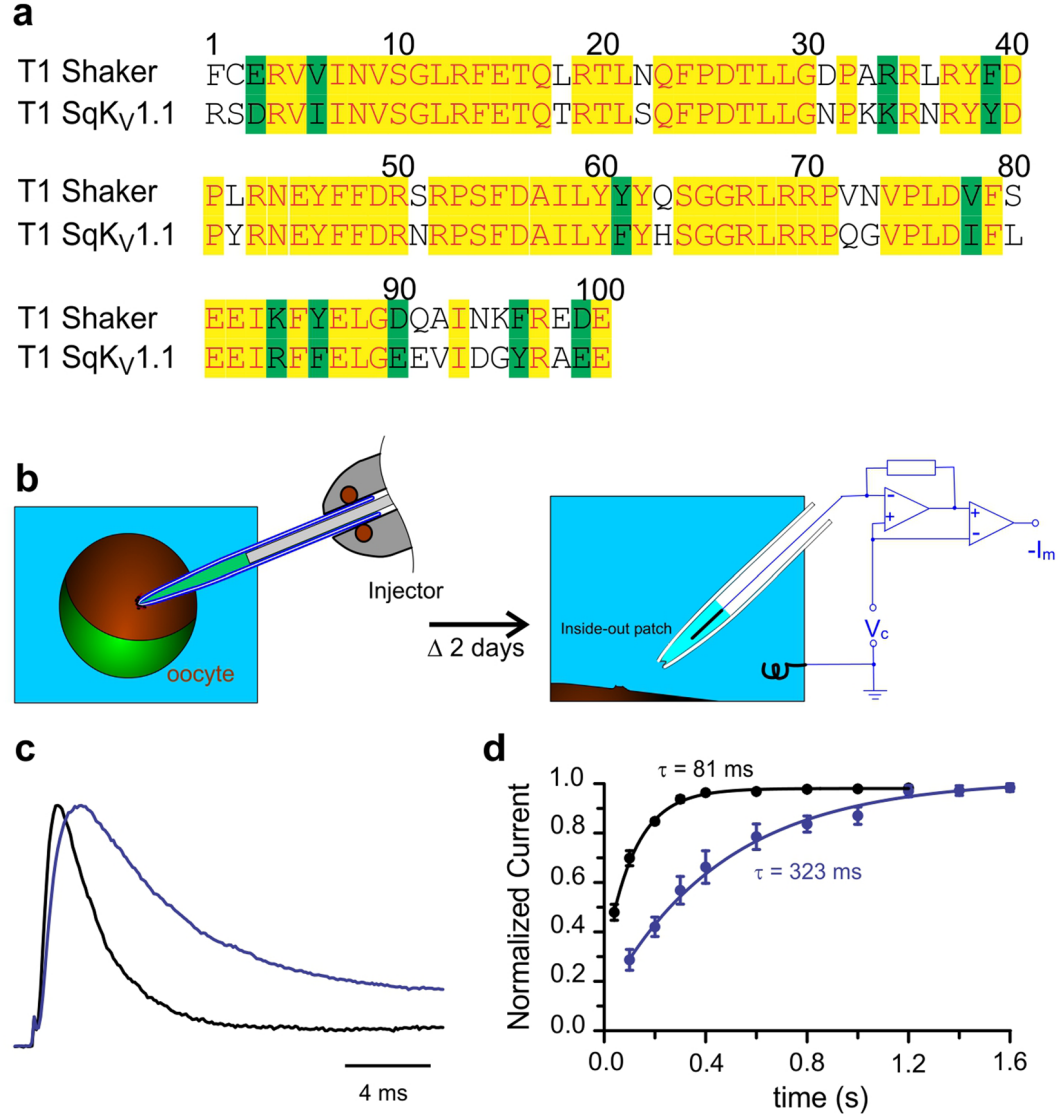
Heterotetramer Shaker K_V_/Sq.K_V_1.1 channel formation. (**a**) T1 domain alignment. Shaker and SqK_V_1.1 T1 domains are similar. Red characters over yellow background represent identical amino acids (71%). Black characters over green background represent conservative substitutions (11%); totaling 82% consensus positions. (**b**) Experimental approach. *Xenopus* oocytes were injected with cRNA. After 2 days incubation, inside-out excised patches were used to assess the potassium current phenotype. (**c)** Potassium current recordings. In black is shown a recording from an oocyte injected only with Shaker K_V_ cRNA, while in blue is shown a recording from an oocyte injected with a mix of cRNAs encoding for Shaker K_V_ and SqK_V_1.1. Both traces were normalized to its respective peak current values. Currents were obtained in response to voltage steps from −70 to + 60 mV. (**d**) Time courses of recovery from inactivation. Recovery was assessed by paired pulse protocols. Activating pulses consisted of 100 ms to + 60 mV, and test pulses were 20 ms long to + 60 mV. Recovery was assessed at −70 mV. Symbols represent the peak current value of the test (second) pulse normalized to the peak current value of its own activating (first) pulse. Solid lines represent single-exponential fits. The time constant for recovery from Shaker K_V_ channels was 81 ms (n = 7), while the time constant corresponding for heterotetramers was 323 ms (n = 9).

Fast inactivation of Shaker K_V_ channels has been shown to be produced by the intracellular N-terminus gate that directly blocks the permeation pathway^49–52^. Since K_V_ channel alpha subunits are known to tetramerize to form functional channels^53^, Shaker K_V_ homotetramers would have four inactivation gates. Nonetheless, it has been demonstrated that only one is sufficient to produce inactivation^54,55^. Newly synthesized Shaker K_V_-like channels in axons inactivate slower than Shaker homotetramers, suggesting that many of these channels might be heterotetrameric channels with less than four inactivation gates. It has been shown earlier that the assembly of distinct alpha subunits to form a functional channel is largely determined by the tetramerization (T1) domain^56,57^. Since *D. gigas* K_V_1.1 and Shaker K_V_ channel T1 domain sequences were found to be highly similar (Fig. 6a), it is plausible that these alpha subunits from different species might co-assemble. To directly test this possibility, we performed experiments in a well-established heterologous system (Fig. 6b). *Xenopus* oocytes were injected with cRNA of Shaker K_V_ channels alone or mixed with cRNA of *D. gigas* K_V_1.1 channels. Figure 6c shows superimposed current recordings from inside-out patches of oocytes injected with only Shaker K_V_ cRNA (black) or the mixture (blue). Interestingly, heterotetrameric channels were seen to recover from inactivation much more slowly (Fig. 6d; blue circles) than the homotetrameric ones (black circles). In fact, channels newly synthesized in axons recovered from inactivation (Fig. 5f) with a time constant comparable to that of heterotetramers in oocytes (Fig. 6d**;** blue circles). Thus, the changes in kinetics of inactivation clearly demonstrated that Squid K_V_1.1 can indeed co-assemble with Shaker K_V_ to form functional heterotetrameric channels.

## Discussion

Much of the axonal electrical signal transmission function has been shown to be manifested at its membrane. The possibility that axons can synthesize membrane proteins locally has been under extensive investigation^1,2,58,59^, as it can provide new mechanistic insights for maintenance of proper axonal function. Here we have shown that the axoplasm of squid giant axon contains a transcriptome representative of a compartment with membrane protein synthesis and trafficking capabilities. Moreover, we have demonstrated not just the presence, but also functional characteristics of membrane proteins, specifically ion channels, newly synthesized within isolated axons.

RNA composition of a subcellular compartment can be indicative of the ongoing cellular processes within the compartment. The giant axon in *D. gigas* offered us an opportunity to examine ~8–10 cm of a singular unmyelinated electrical signaling unit of an axon, utilized immediately after dissection from a freshly captured adult animal (Fig. 1). Pure axoplasm was extruded from a giant axon, thus separated from surrounding Schwann cells, and used for total RNA isolation. Microgram quantities of total RNA was obtained from axoplasm of a single giant axon. The axoplasm was found to contain significant amounts of ribosomal RNA (Fig. 2), an observation which has also been reported earlier in another squid species, *Loligo pealei*^60^. mRNAs encoding β-actin, β-tubulin, neurofilament proteins, enolase and kinesin have been previously reported in squid giant axons^61^. In the present study, deep sequencing of polyadenylated RNA present in a single *D. gigas* giant axon has revealed an exhaustive list of mRNAs that encode many proteins known to be involved in protein synthesis, modification and folding (Supplementary Table S1). This is in agreement with transcriptome studies of the axonal electrical signaling compartment obtained from several other types of neurons, which have shown an mRNA repertoire indicative of axonal translation^30,33–35,62^, thus suggesting that local protein synthesis is likely a fundamental property of axons^1,2,58,63^.

The transcriptome obtained from axons of mammalian spinal cord motoneurons has been reported to contain several membrane protein mRNAs^33^. Similarly, microarray analyses has reported that mRNAs encoding membrane proteins are present in axons obtained from rat dorsal root ganglion neurons (DRGs; sensory neurons)^30^, whereas such mRNAs were largely depleted from mouse DRGs axons when analyzed using deep sequencing^35^. Notably, we found transcripts encoding membrane proteins such as Na^+^/K^+^-ATPase subunits, glutamate receptors, ion channels and transporters in the squid axonal transcriptome (Supplementary Table S1). Even though translation of cytoplasmic proteins from resident mRNAs could be evaluated in squid giant axons using radioactive amino acids in earlier studies, membrane protein synthesis has remained uncertain^14^. During membrane protein synthesis, interaction of the ribosome-nascent chain (RNC) complex with the SRP complex has been shown to be critical for co-translational transport of these polypeptides into ER. The SRP complex, *via* its interaction with the SRP receptor (SR), has been reported to deliver the RNC complex onto a translocon, formed by Sec61 and associated proteins in eukaryotic ER membrane^39^. While polysomes^64^ and smooth ER^65^ have been reported in squid giant axons, it has not been determined whether RNC complexes in axons can bind to ER membranes and deliver the new polypeptide inside the ER for protein folding, modification and assembly. Our RNA-Seq studies showed that the *D. gigas* giant axons contain RNA species representative of the molecular machinery required for RNC delivery to ER during membrane protein synthesis. Firstly, we found enriched amounts of SRP RNA in *D. gigas* giant axons (Fig. 3), which is a RNA species known to be central for assembly of the SRP complex^39^. Consistent with our observations, axons from mice spinal cord motoneurons have been shown to contain twice as much SRP RNA compared to their cell bodies^33^. We have also observed substantial enrichment of RNAs of size similar to SRP RNA in total RNA extracted from a different species of squid, *L. pealei* (data not shown). Secondly, we detected mRNAs encoding protein subunits of SRP complex. Thirdly, substantial amounts of transcripts encoding translocon-forming Sec61α subunit were found present in the RNA obtained from squid giant axons. In addition, a variety of structural and functional ER protein-encoding mRNAs were found in the transcriptome (Supplementary Table S1). Consistent with our work in squid giant axons, immunostaining studies in mammalian axons has shown the presence of ER-associated proteins like Sec61, SRP54, protein disulphide isomerase, as well as Golgi-associated proteins such as GM130, Giantin, TGN46 amongst others, which are indicative of the presence of endomembranous structures in mammalian axons^41,43^. Recently, extensive axonal endomembrane organelles have been described in mammalian neurons^66^.

The functionality of axonal membrane protein synthesis machinery can be validated by direct measurement of function of newly synthesized membrane proteins at the axolemma of excised axons. Several studies have utilized different strategies to monitor the synthesis of membrane proteins in axons. In *L. pealei* giant axons, membrane protein synthesis has been studied using radioactive amino acids, but suffered a major limitation that the signal from the membrane could not be resolved from the signal originating from the surrounding Schwann cells^14^. Biochemical measurements to follow local translation of glycosyl phosphatidylinositol (GPI)-anchored placental alkaline phosphatase^21^ or monitoring changes in membrane excitability due to signaling through newly synthesized conopressin receptor in *Aplysia* axons^44^, offered indirect ways to assay function of an integral membrane protein. Therefore, direct measurement of a membrane protein in a mature axon’s electrical signaling compartment has persisted to be technically challenging.

The design of our functional experiments circumvented these difficulties in obtaining an unequivocal proof for synthesis of membrane proteins in axons and their delivery to the membrane. Potassium channels are known to be essential components of an axon membrane. We used two different potassium channels: *D. gigas* K_V_1.1 (homologous) and *Drosophila* Shaker K_V_ channel (heterologous). Measurement of current flow and kinetics under voltage clamp provided a direct readout of the function of both these integral membrane proteins. Shaker channel mRNAs are known to get translated as monomeric subunits whose assembly into functional tetrameric channels requires processing in the ER-Golgi system^67^. Therefore, a positive current signal from a functional Shaker channel in axolemma would serve as a strong indication of the presence of a functional endomembrane machinery which can be used for membrane protein processing. In addition, Shaker K_V_ channel has been shown to exhibit distinct inactivation kinetics, which were absent in squid K_V_1.1 channel under voltage clamp^68^. Different kinetics of the two channels were used in our experiments to distinguish the heterologous channel from the homologous one. Because of the placement of the electrodes across the axolemma, only the signal originating from the ion channels incorporated in the axolemma, without contamination from either intra-axonal membranes or surrounding Schwann cells, could be recorded with high confidence. When isolated squid giant axons were injected with *in vitro* synthesized RNA encoding homologous *D. gigas* K_V_ channel, an increase in K^+^ current was observed (Fig. 5). When axons were injected with *in vitro* transcribed RNA encoding the Shaker K_V_ channel, along with an increase in current, channels were found to exhibit inactivation characteristics, typical of this type of channel subunits (Fig. 5). This provided evidence that excised axons from adult animals, immediately used after dissection, without culturing, have the machinery which can utilize a polyadenylated, capped mRNA to translate a polypeptide, fold and assemble it, and target it to the axon membrane as a functional membrane protein. Translation of cytoplasmic proteins in squid axons has been reported to be sensitive to external application of cycloheximide, a translation inhibitor^14^. However, internal delivery of cycloheximide to monitor decay in current and differential kinetics was technically not feasible after the axon was already clamped. Therefore, our study relied largely on appearance of inactivation kinetics in the potassium current across axolemma.

Interestingly, we found that the recovery from inactivation kinetics for the new channels, resembled the potassium channels containing less than four identical Shaker channel subunits^54,55,69^. We have presented evidence that the Shaker channel subunits heterotetramerize with the squid K_V_ channel subunits in *Xenopus* oocytes (Fig. 6). This implied that all native squid K_V_ channel subunits present in the axoplasm of isolated axons are not found as fully assembled potassium channels. But some population would be available as unassembled subunits in the squid giant axons, possibly in ER-like compartments, which can heterotetramerize with the newly synthesized Shaker K_V_ channel subunits.

Overall our results have provided definitive evidence that isolated axons can utilize resident machinery to synthesize fully functional membrane proteins such as ion channels containing multiple transmembrane segments. Our discoveries have established a powerful axonal heterologous expression system which can be exploited to further explore detailed molecular mechanisms regulating axonal membrane proteome, at different levels such as protein translation, post-transcriptional and posttranslational modifications, which could have important implications in axonal function.

## Methods

### Specimen collection and dissection

*D. gigas* were caught from the Pacific waters near the Montemar campus, University of Chile. Squids were decapitated immediately, and their heads and mantle were stored in cold sea water. These were transported to the laboratory within 1 hour. The mantle was used for obtaining the stellate ganglia and giant axons. The axons were immediately separated from the ganglion, cleaned and placed on an RNase-free surface. The axoplasm was extruded from the axonal sheath using a roller and collected directly in an RNase-free microcentrifuge tube. The axonal sheath contains a single layer of periaxonal Schwann cells.

### Total RNA isolation and analysis

Stellate ganglia samples were homogenized in Trizol using a mortar-pestle. Axonal samples were homogenized in Trizol using Kontes pellet pestles (Kimble Chase). RNA purification was carried out using PureLink RNA Mini kit (Ambion) as per manufacturer’s protocol (for axonal samples, 0.2 μg/μl glycogen was added to the sample before loading it onto the spin column). ~3 μg total RNA was obtained from the axoplasm. Total RNA from all samples were analyzed using Agilent Bioanalyzer 2100. RNA from all samples were run using the RNA 6000 Nano kit.

Squid rRNA sequences were found to be similar to mouse rRNA sequences. Therefore, RiboZero rRNA Removal kit (Human/Rat/Mouse) (Illumina) was used for rRNA depletion of 1 μg axonal sample. The depleted RNA was run using RNA 6000 Pico kit in the Agilent Bioanalyzer 2100.

### Library construction, sequencing and quality control

Poly(A)-enriched libraries were constructed for one axoplasm sample and one axon sample using Truseq Stranded mRNA RNA Library Prep kit (Illumina) according to manufacturer’s protocol. Ribo-depleted library was separately constructed using the Truseq Stranded Total RNA with Ribo-Zero Globin kit (Illumina), according to manufacturer’s protocol with the following alteration: the ribo-depleted library was not subjected to fragmentation before sequencing. This was done to ensure enrichment of signal from species under peak (iii) over and above the longer RNA.

All libraries prepared were paired-end sequenced 125 bp per read via HiSeq. 2000 (Illumina). Target sequencing depth was >120 M read pairs for each of the poly(A)-enriched libraries and >50 M read pairs for the ribo-depleted library. Post sequencing, reads per library were quality inspected using the “FastQC” tool (https://www.bioinformatics.babraham.ac.uk/projects/fastqc/), adaptor clipped using the “fastx_clipper” tool (http://hannonlab.cshl.edu/fastx_toolkit/commandline.html#fastx_clipper_usage), hard quality-trimmed (-Q33 -f 18 -l 123) using the “fastx_trimmer” tool (http://hannonlab.cshl.edu/fastx_toolkit/commandline.html#fastx_trimmer_usage), and dynamic quality-trimmed (-Q33 -q 20) using the “fastq_quality_filter” tool (http://hannonlab.cshl.edu/fastx_toolkit/commandline.html#fastq_quality_filter_usage). Intact read-pairs remaining post clipping and trimming were subset from orphan reads using common awk commands (http://www.grymoire.com/Unix/Awk.html).

### *De novo* assembly, map back and ORF determination

Intact read pairs generated after quality control from the two poly(A)-enriched libraries were combined and passed to the “De Novo Assembly” tool supported in the CLC Genomics Workbench (https://www.qiagenbioinformatics.com/products/clc-genomics-workbench/) using select parameters (Word size = 20, Bubble size = 50, Contig length = 300, Perform scaffolding = True). Component sequences that returned were then passed to the “Find Open Reading Frames (ORFs)” tool supported in the CLC Genomics Workbench using select parameters (Use all start codons in genetic code = True, Search both strands = True, Allow for open-ended sequence = True, Minimum length = 100 codons, Annotate stop codons = True). Identified ORFs per component were then characterized by “blastx” (https://www.ncbi.nlm.nih.gov) using reviewed protein sequences only found in the “Swiss-Prot” database of “UniProt” (http://www.uniprot.org/); unreviewed sequences were not used. Annotation of ORFs included the assignment of the “Uniprot” accession with the highest returned “Score” by “blastx” having an “E-value” <1e-6. ORFs were not annotated if the “E-value” returned by blastx was >= 1e-6. These annotations represented the orthologs of proteins detected from RNA-seq data. Post annotation, enumerated expression per ORF in “Reads Per Kilobase Million” mapped reads units, or “RPKM”, was calculated after performing map back of the intact read pairs per poly(A)-enriched library against the components. Map back was accomplished using the “Map Reads to Reference” tool supported in the CLC Genomics Workbench in conjunction with select parameters (Match score = 1, Mismatch cost = 2, Linear gap cost = True, Insertion cost = 3, Deletion cost = 3, Length fraction = 0.9, Similarity fraction = 0.9, Auto-detect paired distances = True, Map non-specific reads randomly = True).

Intact read pairs obtained from the ribo-depleted library were used to generate a separate independent set of components using Trinity (https://github.com/trinityrnaseq/trinityrnaseq/wiki) under select parameters (–seqType fq–max_memory 50 G–CPU 14–SS_lib_type FR–min_contig_length 200–min_kmer_cov 2–KMER_SIZE 25–max_reads_per_graph 200000–min_glue 2–min_iso_ratio 0.05–glue_factor 0.05–group_pairs_distance 500–path_reinforcement_distance 75–min_per_id_same_path 98–max_diffs_same_path 2–max_internal_gap_same_path 10) for de novo assembly and Bowtie2 (http://bowtie-bio.sourceforge.net/bowtie2/index.shtml) for map back (–local). Component sequences were blastn (National Center for Biotechnology Information (NCBI)) characterized.

Raw sequence files per library are available for download through the NCBI Short Read Archive (SAMN08026329, SAMN08026330, SAMN08026331). Supplementary files, including the component sequences per assembly and ORF blast annotations, are available for download at https://data.ninds.nih.gov/Holmgren/MathurC/.

### Ingenuity Pathway Analysis

A dataset of unique proteins which had an RPKM value within the top 25^th^ percentile amongst the predicted axonal ORFs was determined. The Uniprot accession numbers for all these proteins were submitted for core analysis using Ingenuity Pathway Analysis software (Qiagen). The categories representing various molecular and cellular functions were considered for further analysis. The software provides a p-value depending on the number of focus genes in the dataset and total number of genes enlisted for the category in the database. This p-value was used as a representation to determine the cellular processes that are crucial for axonal function.

### Expression and electrophysiology using squid giant axons

*D. gigas* K_V_1.1 channel was subcloned in a pGemSqHE vector in which the 5’ and 3’ UTR sequences of the squid Na^+^/K^+^-ATPase beta subunit flank the channel’s coding region. These UTR regions were selected because they belong to one of the most abundant mRNA species of a membrane protein in the axoplasm’s sample. Shaker K_V_ channel was subcloned in pGemHE vector. cDNAs were linearized with NheI and RNAs were synthesized using T7 RNA polymerase (Ambion). RNA was injected in a solution containing (mM): 5 GTP sodium, 5 ATP magnesium, 50 K glutamate, 50 KPO_4_, 5 K phospho-arginine, 5 K Hepes and 5 Mg Hepes (pH~ 7.5–8). Axons were mounted in a three-compartment chamber (Fig. 1b) to record currents from the central compartment using a virtual ground current-to-voltage converter using the lateral compartments as guards, connected to ground. A 100 µm platinized platinum wire was inserted from the right side of the chamber along the axis of the axon and from the other side a cannula of about 80 µm diameter filled with 0.6 M KCl was inserted to measure the internal potential. The cannula contained a floating 25 µm platinum wire to increase high frequency response. The external potential was measured in the central external compartment with a cannula filled with 3 M KCl in agar. The custom-built voltage clamp used high speed operational amplifiers and could settle the membrane potential in less than 10 µs. A custom written program controlled a USB-1604-2AO acquisition unit (Measurement Computing) that delivered command voltages by a 16-bit D/A converter and acquired current and voltage with two separate 16-bit A/D converters sampling at 1 to 5 µs per point. Current was low pass filtered at ¼ of the sampling frequency by an 8-pole Bessel filter before it was fed into the A/D converter. The acquired data was analyzed with a custom written program. The external solution used was filtered (0.22 μm) sea water with 20 μM tetrodotoxin. Measurements of currents under voltage clamp were done immediately after the axon was mounted in the chamber and then at intervals of about 30–60 minutes. In between measurements the voltage clamp was turned off. The temperature of the chamber was about 24 °C during all the recordings.

### Expression and electrophysiology using Xenopus oocytes

Same RNAs used in axons were injected into *Xenopus laevis* oocytes. Shaker K_V_ channels were found to be more efficiently translated by oocytes than *D. gigas* K_V_1.1 channels. Therefore, we used ratios of 1:5 (Shaker:*D. gigas*) or higher when both constructs were mixed. Defolicullated oocytes were obtained from Ecocyte Bioscience (Austin, TX) and microinjected with 50 nl of RNA mix containing 5–10 ng of Shaker Kv RNA, 25–50 ng of *D. gigas* Kv1.1 RNA or the mix of both. After injection, oocytes were incubated at 17 °C in ND96 medium. Electrophysiological experiments were performed 1–2 days after injection. The oocytes’ vitelline layer was manually removed. Excised inside-out patches were obtained with borosilicate pipettes of 2 MΩ. Current acquisition was performed with a Digidata 1322 A (Molecular Devices, Sunnyvale, Ca), an Axopatch 200b patch clamp amplifier (Molecular Devices) and Clampex Software (Molecular Devices). The Bath solution contained (in mM) 160 KCl, 0.5 MgCl_2_, 1 EGTA and 10 HEPES (pH 7.4). The pipette solution contained (in mM) 150 NaCl, 10 KCl, 3CaCl_2_, 1 MgCl_2_ and 10 HEPES (pH 7.4). All recordings were made at room temperature.

## Electronic supplementary material

Supplementary Table S1
